# A Rate-Reduced Neuron Model for Complex Spiking Behavior

**DOI:** 10.1186/s13408-017-0055-3

**Published:** 2017-12-11

**Authors:** Koen Dijkstra, Yuri A. Kuznetsov, Michel J. A. M. van Putten, Stephan A. van Gils

**Affiliations:** 1 Department of Applied Mathematics, University of Twente, Enschede, The Netherlands; 2 Department of Mathematics, Utrecht University, Utrecht, The Netherlands; 3 Department of Clinical Neurophysiology, University of Twente, Enschede, The Netherlands; 4 Department of Clinical Neurophysiology, Medisch Spectrum Twente, Enschede, The Netherlands

## Abstract

We present a simple rate-reduced neuron model that captures a wide range of complex, biologically plausible, and physiologically relevant spiking behavior. This includes spike-frequency adaptation, postinhibitory rebound, phasic spiking and accommodation, first-spike latency, and inhibition-induced spiking. Furthermore, the model can mimic different neuronal filter properties. It can be used to extend existing neural field models, adding more biological realism and yielding a richer dynamical structure. The model is based on a slight variation of the Rulkov map.

## Introduction

Networks of coupled neurons quickly become analytically intractable and computationally infeasible due to their large state and parameter spaces. Therefore, starting with the work of Beurle [1], a popular modeling approach has been the development of continuum models, called neural fields, that describe the average activity of large populations of neurons (Wilson and Cowan [2, 3], Nunez [4], Amari [5, 6]). In neural field models, the network architecture is represented by connectivity functions and the corresponding transmission delays, while differential operators characterize synaptic dynamics. All intrinsic properties of the underlying neuronal populations are condensed into firing rate functions, which replace individual neuronal action potentials and map the sum of all incoming synaptic currents to an outgoing firing rate. While some neural field models incorporate spike-frequency adaptation (Pinto and Ermentrout [7, 8], Coombes and Owen [9], Amari [10, 11]), more complex spiking behavior such as postinhibitory rebound, phasic spiking and accommodation, first-spike latency, and inhibition-induced spiking is mostly absent, an exception being the recent reduction of the Izhikevich neuron (Nicola and Campbell [12], Visser and van Gils [13]).

Here, we present a rate-reduced model that is based on a slight modification of the Rulkov map (Rulkov [14], Rulkov et al. [15]), a phenomenological, map-based single neuron model. Similar to Izhikevich neurons (Izhikevich [16]), the Rulkov map can mimic a wide variety of biologically realistic spiking patterns, all of which are preserved by our rate formulation. The rate-reduced model can therefore be used to incorporate all the aforementioned types of spiking behavior into existing neural field models.

This paper is organized as follows. In Sect. 2, we present the single spiking neuron model our rate-reduced model is based upon, and illustrate different spiking patterns and filter properties. In Sect. 3 we heuristically reduce the single neuron model to a rate-based formulation, and show that the rate-reduced model preserves spiking and filter properties. We give an example of a neural field that is augmented with our rate model in Sect. 4 and end with a discussion in Sect. 5.

## Single Spiking Neuron Model

In this section we present a phenomenological, map-based single neuron model, which is a slight modification of the Rulkov map (Rulkov [14], Rulkov et al. [15]). The Rulkov map was designed to mimic the spiking and spiking-bursting activity of many real biological neurons. It has computational advantages because the map is easier to iterate than continuous dynamical systems. Furthermore, as we will show in this paper, it is straightforward to obtain a rate-reduced version of a slightly modified version of the Rulkov model.

The Rulkov map consists of a fast variable *v*, resembling the neuronal membrane potential, and a slow adaptation variable *a*. In our modification of the original model, the adaptation only implicitly depends on the membrane potential through a binary spiking variable. As we will show in the next section, this modification allows for an easy decoupling of the membrane potential and adaptation variable, and therefore a straightforward rate reduction of the model. The cost of the modification is the loss of subthreshold oscillation dynamics. The modified Rulkov map is given by

SNM $$ \textstyle\begin{cases} v_{n+1} = f(v_{n}, v_{n-1},\kappa u_{n}-a_{n}-\theta), \\ a_{n+1} = a_{n}-\varepsilon(a_{n}+(1-\kappa)u_{n}-\gamma s_{n}), \end{cases} $$

where the piecewise continuous function $f\colon\mathbb{R}^{3}\to \mathbb{R}$ is given by

1 $$ f(x_{1},x_{2},x_{3})= \textstyle\begin{cases}\frac{2500+150x_{1}}{50-x_{1}}+50x_{3} &\text{if } x_{1}< 0, \\ 50+50x_{3} &\text{if } 0\leq x_{1}< 50+50x_{3} \quad\wedge\quad x_{2}< 0, \\ -50 &\text{otherwise}. \end{cases} $$

The form of *f* is chosen to mimic the shape of neuronal action potentials. The variable *u* in (SNM) represents external (synaptic) input to the cell, which we assume to be given, and *s* is a binary indicator variable, given by

2 $$ s_{n} = \textstyle\begin{cases} 1&\text{if the neuron spiked at iteration $n$,} \\ 0 &\text{otherwise.} \end{cases} $$

A Rulkov neuron spikes at iteration *n* if its membrane potential is reset to $v_{n+1}=-50$ in the next iteration. It follows from (1) that the spiking condition in (2) is satisfied if and only if

3 $$ v_{n}\geq0\quad\wedge\quad \bigl(v_{n}\geq50+50(\kappa u_{n}-a_{n}-\theta ) \quad\vee\quad v_{n-1}\geq0 \bigr). $$

The dependence of $v_{n+1}$ on $v_{n-1}$ in (SNM) ensures that a neuron always spikes if its membrane potential is non-negative for two consecutive iterations, independent of the external input *u*. To mimic spiking patterns of real biological neurons, one time step should correspond to approximately 0.5 ms of time.

The parameter $0<\varepsilon<1$ in (SNM) sets the time scale of the adaptation variable and *γ* determines the adaptation strength. The parameter *θ* can be interpreted as a spiking threshold: for constant external input $u_{n}=\varphi$, the neuron spikes persistently if and only if $\varphi>\theta$. After a change of variable $a_{n}\rightarrow a_{n}+(1-\kappa)\varphi$ and parameter $\theta\rightarrow\theta-\varphi$, constant external input vanishes. Therefore, the asymptotic response to constant input does not depend on the parameter *κ*. However, the parameter *κ* can be used to tune the transient response of the neuron to changes in external input, as it determines how input is divided between the fast and the slow subsystem of (SNM). For parameter values $\kappa \in[0,1]$, *κ* can be interpreted as the fraction of the input that is applied to the fast subsystem, and therefore determines (together with *ε*) how quickly the membrane potential dynamics react to changes in input. Since the effective drive of the system is given by $\kappa u_{n}-a_{n}$, changes in external input are initially magnified for $\kappa>0$. Asymptotically, this is then counterbalanced by additional adaption. Finally, for $\kappa<0$, the initial response of the membrane potential to a change in input is reversed, i.e. an increase in external input initially has an inhibitory effect, and a decrease in external input initially has an excitatory effect.

### Fast Dynamics

The Rulkov map (SNM) with $0<\varepsilon\ll1$ is a slow-fast system, and we can explore the fast spiking dynamics of the model by assuming the suprathreshold drive $\kappa u_{n}-a_{n}-\theta =\varsigma$ is constant. In this case, (SNM) reduces to the fast subsystem

FSS $$ v_{n+1}= \textstyle\begin{cases}\frac{2500+150v_{n}}{50-v_{n}}+50\varsigma& \text{if } v_{n}< 0, \\ 50+50\varsigma& \text{if } 0\leq v_{n}< 50+50\varsigma, \\ -50 & \text{otherwise}. \end{cases} $$

**Fig. 1:**
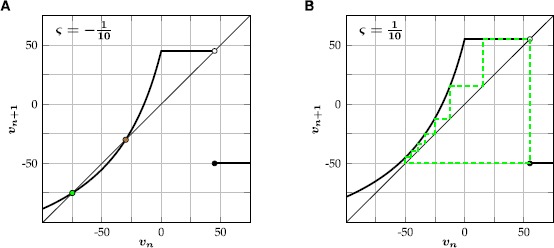
Illustration of the fast subsystem (FSS) of (SNM). **(** **A** **)** For $\varsigma=-\tfrac{1}{10}$ there exist a stable (*green*) and unstable (*orange*) fixed point. **(** **B** **)** For $\varsigma=\tfrac{1}{10}$ the system will settle into a stable periodic orbit (*dashed green line*) with period $P (\tfrac{1}{10} )=8$

The map (FSS) undergoes a saddle-node bifurcation at $\varsigma=0$ (Fig. 1). For $\varsigma<0$ there exist a stable and an unstable fixed point, given by

4 $$ v_{\mathrm{s}}=25 \bigl(\varsigma-2-\sqrt{\varsigma^{2}-8\varsigma } \bigr), \qquad v_{\mathrm{u}}=25 \bigl(\varsigma-2+\sqrt{\varsigma ^{2}-8\varsigma} \bigr), $$

respectively (Fig. 1A), while the system will settle into a stable periodic orbit for $\varsigma>0$ (Fig. 1B). In the former case the unstable fixed point acts as an excitation threshold: if the value of the membrane potential exceeds this point, it will spike once and then decay back to the stable equilibrium. Since the unstable fixed point $v_{\mathrm{u}}$ always lies to the right of the ‘reset potential’ $v=-50$, a stable fixed point and a periodic orbit can never coexist. This guarantees that we can define a firing rate function $S\colon\mathbb{R}\to\mathbb{Q}$ for the fast subsystem (FSS), given by

5 $$ S(\varsigma)= \textstyle\begin{cases} 0 & \text{for } \varsigma\leq0,\\ \frac{1}{P(\varsigma)} & \text{for } \varsigma>0, \end{cases} $$

where $P\colon\mathbb{R}_{>0}\to\mathbb{N}$ maps the drive to the period of the corresponding stable limit cycle of (FSS). The fast subsystem (FSS) is piecewise-defined on the ‘left’ interval $(-\infty,0)$, the ‘middle’ interval $[0,50+50\varsigma)$, and the ‘right’ interval $[50+50\varsigma,\infty)$. The left interval is mapped to the left and middle interval, and the middle and right interval are mapped to right and left interval, respectively. The period of a limit cycle of (FSS) therefore only depends on the number of iterations in the left interval. Note, however, that the shape of the function *f* given in (1) can easily be changed to support bistability in the fast subsystem, which allows for some additional dynamics such as ‘chattering’, a response of periodic bursts of spikes to constant input (Rulkov [14]).

### Spiking Patterns

Izhikevich [17] classified different features of biological spiking neurons, most of which can be mimicked by our modified Rulkov model (SNM). In the following, we discuss the role of the model parameters with the help of a few physiologically relevant examples.

#### Tonic Spiking/Fast Spiking

**Fig. 2:**
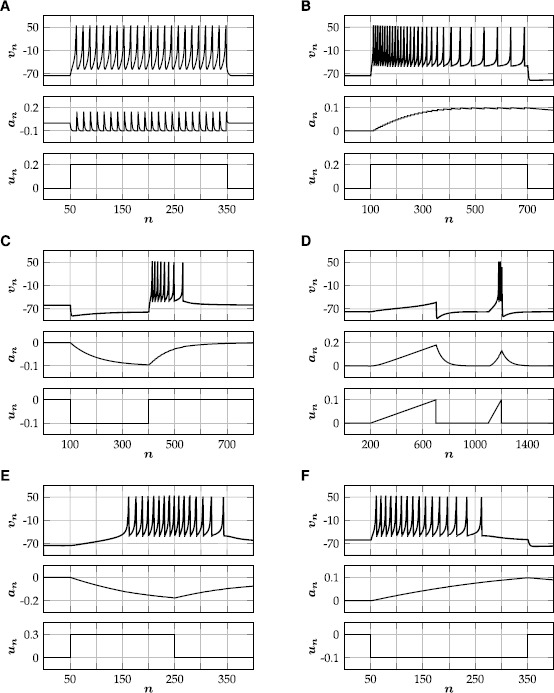
Different types of spiking patterns generated by the single neuron model (SNM). Corresponding parameter values $(\theta,\kappa,\varepsilon,\gamma )$ are given in brackets. **(** **A** **)** Tonic spiking $(\tfrac{1}{10},\tfrac{1}{2},\tfrac{1}{2},\tfrac{1}{2} )$. **(** **B** **)** Spike-frequency adaptation $(\tfrac{1}{10},1,\tfrac{1}{1000},5 )$. **(** **C** **)** Rebound spiking $(\tfrac{1}{50},2,\tfrac{1}{100},\frac{1}{5} )$. **(** **D** **)** Accommodation $(\tfrac{3}{25},3,\tfrac{1}{50},\tfrac{2}{5} )$. **(** **E** **)** Spike latency $(\tfrac{1}{10},0,\tfrac{1}{200},\tfrac{2}{5} )$. **(** **F** **)** Inhibition-induced spiking $(\tfrac{1}{50},-1,\tfrac{1}{500},\tfrac{2}{5} )$

Tonically spiking (also called ‘fast spiking’) neurons respond to a step input with spike trains of constant frequency. Most inhibitory neurons are fast spiking (Izhikevich [17]). In the modified Rulkov model this can be achieved by choosing a ‘large’ $(1>\varepsilon>\frac{1}{10} )$ value for the time scale parameter, in which case the influence of a single spike on the adaptation variable decays very fast. Therefore, the value of the adaptation variable is dominated by the timing of the last spike and the influence of older spikes is negligible (Fig. 2A). Since the time scale separation is small, the qualitative dynamics does not depend on *κ*.

#### Spike-Frequency Adaptation/Regular Spiking

Most cortical excitatory neurons are not ‘fast spiking’, but respond to a step input with a spike train of slowly decreasing frequency, a phenomenon known as ‘spike-frequency adaptation’ (also called ‘regular spiking’). This kind of spiking behavior can be modeled by applying all input to the fast subsystem ($\kappa=1$) and choosing $\varepsilon\ll 1$. The adaptation variable then acts as a slow time scale, such that a single spike has a long-lasting effect on the adaptation variable (Fig. 2B). The level of adaptation can be controlled with *γ*.

#### Rebound Spiking and Accommodation

The excitability of some neurons is temporarily enhanced after they are released from hyperpolarizing current, which can result in the firing of one or more ‘rebound spikes’. Rebound spiking is an important mechanism for central pattern generation for heartbeat and other motor patterns in many neuronal systems (Chik et al. [18]). In the modified Rulkov map, postinhibitory rebound spiking can be modeled by choosing $\kappa>1$. In this case, the adaptation variable will become negative while the cell gets hyperpolarized, which can be sufficient to trigger temporary spiking once the inhibitory input is turned off (Fig. 2C). Similarly, excitatory ‘subthreshold’ ($u_{n}<\theta$) input can elicit temporary spiking if the input is ramped up sufficiently fast (Fig. 2D).

#### Spike Latency and Inhibition-Induced Spiking

If all input is applied to the slow subsystem ($\kappa=0$), there can be a large latency between the input onset and the first spike of the neuron, yielding a delayed response to a pulse input (Fig. 2E). For $\kappa<0$, the initial response of the model to changes in input is reversed: excitation initially leads to hyperpolarization of the neuron and inhibition can induce temporary spiking (Fig. 2F). This inhibition-induced spiking is a feature of many thalamo-cortical neurons (Izhikevich [17]).

### Neuronal Filtering

In the previous section, we illustrated how the parameter *κ* can tune transient spiking responses of the modified Rulkov map to changes in external input. In reality, neurons often receive strong periodic input, e.g. from a synchronous neuronal population nearby. Information transfer between neurons may be optimized by temporal filtering, which is especially important when the same signal transmits distinct messages (Blumhagen et al. [19]). In this section, we study the response of (SNM) to harmonic input

6 $$ u_{n}= \varphi\cos \biggl(\frac{\omega\pi n}{1000}+\vartheta \biggr), $$

with amplitude *φ*, phase shift $\vartheta\in[0,2\pi)$, and where $\omega\in[0,1000]$ corresponds to the input frequency in Hz assuming that one iteration of (SNM) corresponds to 0.5 ms of time. A Rulkov neuron (SNM) will never spike if

7 $$ \kappa u_{n}-a_{n}\leq\theta \quad\forall n. $$

In this case, the adaptation reduces to the simple linear equation

8 $$ a_{n+1} = (1-\varepsilon)a_{n}-\varepsilon(1- \kappa)u_{n}, $$

with explicit solution

9 $$ a_{n} = -\varepsilon(1-\kappa)\sum _{m=1}^{\infty }(1-\varepsilon)^{m-1}u_{n-m}. $$

Inserting (6) into (9) now yields

10 $$ \begin{aligned}[b] \kappa u_{n}-a_{n}&= \kappa\varphi\cos \biggl(\frac{\omega\pi n}{1000}+\vartheta \biggr)\\ &\quad {}+\varepsilon(1- \kappa)\varphi\sum_{m=1}^{\infty}(1- \varepsilon)^{m-1}\cos \biggl(\frac{\omega\pi (n-m)}{1000}+\vartheta \biggr) \\ &=F(\omega)\frac{\varphi}{2}e^{(\frac{\omega\pi n}{1000}+\vartheta )i}+\overline{F(\omega)} \frac{\varphi}{2}e^{-(\frac{\omega\pi n}{1000}+\vartheta)i} \\ &=\bigl\lvert F(\omega)\bigr\rvert \varphi\cos \biggl(\frac{\omega\pi n}{1000}+ \vartheta+\arg \bigl(F(\omega) \bigr) \biggr), \end{aligned} $$

where the overline denotes complex conjugation and the frequency response $F\colon[0,1000]\mapsto\mathbb{C}$ is given by

11 $$ F(\omega)=\kappa+\frac{\varepsilon(1-\kappa)}{e^{\frac{\omega \pi i}{1000}}+\varepsilon-1}. $$

The absolute value and argument of the frequency response determine the relative magnitude and phase of the output, respectively. It follows that a Rulkov neuron (SNM) receiving periodic input (6) does not spike if

12 $$ \bigl\lvert F(\omega)\bigr\rvert \varphi\leq\theta. $$

The inverse statement is not true, even if *ω* and *ϑ* in (6) are chosen such that

13 $$ \cos \biggl(\frac{\omega\pi n}{1000}+\vartheta+\arg \bigl(F(\omega ) \bigr) \biggr)=1 \quad\text{for some }n\in\mathbb{N}. $$

**Fig. 3:**
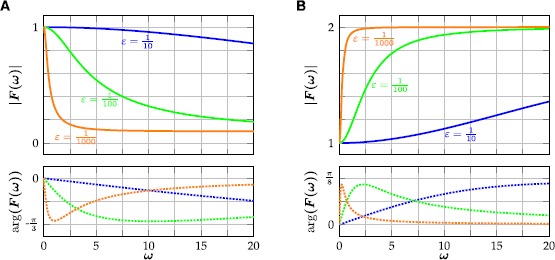
Illustration of the frequency response (11) for different values of *ε*. **(** **A** **)** For $\kappa=\tfrac{1}{10}$ high frequencies get attenuated. **(** **B** **)** For $\kappa=2$ high frequencies get amplified. Note the similarity, which is caused by the fact that $F(\omega)-1$ is an odd function of $1-\kappa$

Since it can take a few iterations of the map to converge to its periodic orbit, a neuron will only spike if its drive is larger than the threshold *θ* for a sufficiently long time. The modulus of the frequency response (11) is given by

14 $$ \bigl\lvert F(\omega) \bigr\rvert =\sqrt{\frac{\varepsilon^{2}+2\kappa (\kappa-\varepsilon ) (1-\cos (\frac{\omega\pi }{1000} ) )}{\varepsilon^{2}+2 (1-\varepsilon ) (1-\cos (\frac{\omega\pi}{1000} ) )}}, $$

and it follows that $\lvert F\rvert$ is strictly decreasing if and only if $\kappa\in(-1+\varepsilon,1)$, and increasing otherwise (Fig. 3). Clearly,

15 $$ F(0)=1, \qquad F(1000)=\frac{2\kappa-\varepsilon}{2-\varepsilon}. $$

**Fig. 4:**
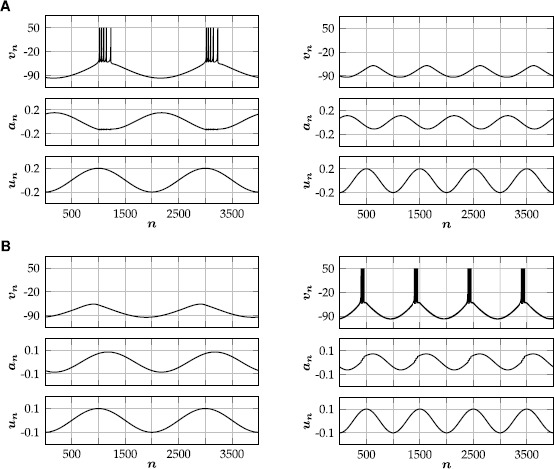
Responses of (SNM) to periodic input, illustrating neuronal filter properties. **(** **A** **)** For $\kappa=\tfrac{1}{10}$ the neuron acts as a low-pass filter. Input with an amplitude of $\varphi=\tfrac{1}{5}$ elicits a spiking response for $\omega=1$, whereas the neuron is quiescent for $\omega=2$. **(** **B** **)** For $\kappa=2$, the neuron acts as a high-pass filter. Input with amplitude $\varphi=\tfrac{1}{10}$ elicits a spiking response for $\omega=2$, whereas a lower input frequency of $\omega=1$ does not. In both examples, $(\theta,\varepsilon,\gamma )= (\tfrac{1}{7},\tfrac{1}{200},2 )$

The input parameter *κ* can therefore be used to model filter properties of the neuron. For $-1+\varepsilon<\kappa<1$ high frequencies get attenuated and a neuron can act as a low-pass filter in the sense that periodic input within a certain amplitude range only elicits a spiking response if its frequency is low enough (Fig. 4A). Similarly, for $\kappa>1$ (and $\kappa<-1+\varepsilon$), high frequencies get amplified and there exists an amplitude range for which the neuron acts as a high-pass filter (Fig. 4B).

## The Rate-Reduced Neuron Model

Neural field models are based on the assumption that neuronal populations convey all relevant information in their (average) firing rates. If one wants to incorporate certain spiking dynamics, one has to come up with a corresponding rate-reduced formulation first. In this section we present a rate-reduced version of the Rulkov model (SNM) that can be used to extend existing neural field models.

The adaptation variable *a* in the spiking neuron model (SNM) only implicitly depends on the membrane potential *v* via the binary spiking variable *s*. We can therefore decouple the adaption variable from the membrane potential by replacing the binary spiking variable defined in (2) by the instantaneous firing rate (5) of the fast subsystem (FSS), yielding

16 $$ a_{n+1} = a_{n}-\varepsilon \bigl(a_{n}+(1-\kappa)u_{n}-\gamma S(\kappa u_{n}-a_{n}-\theta) \bigr). $$

By interpreting (16) as the forward discretization of an ordinary differential equation, we arrive at the continuous time rate-reduced model

RNM $$ \frac{1}{\varepsilon}\frac{\mathrm{d}a}{\mathrm{d}t} = -a-(1-\kappa)u+ \gamma S(\kappa u-a-\theta). $$

**Fig. 5:**
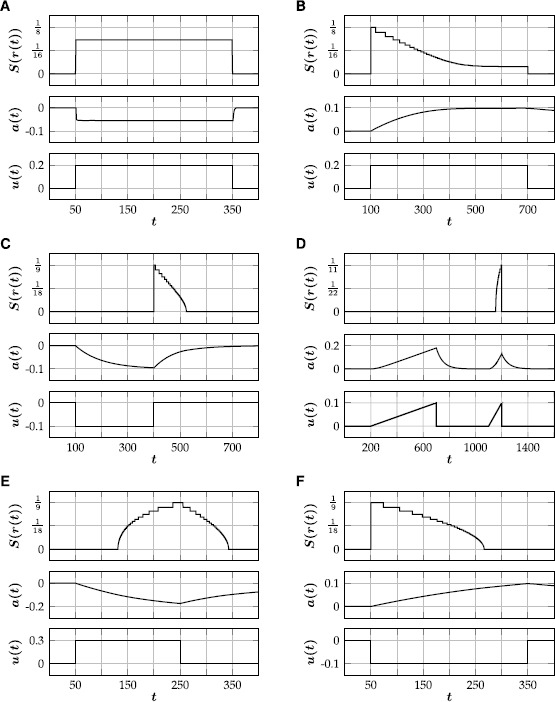
Different types of spiking behavior generated by the rate-reduced model (RNM). Top traces show the firing rate with $r(t)=\kappa u(t)-a(t)-\theta$. Corresponding parameter values $(\theta,\kappa,\varepsilon,\gamma)$ are given in brackets. For small values of *ε* (i.e. a large time scale separation), there is excellent agreement with the corresponding examples of the full model (Fig. 2), which is quantified by comparing the integral of the spiking rate in the reduced model to the number of spikes in the full model. **(** **A** **)** Tonic spiking $(\frac{1}{10},\tfrac{1}{2},\tfrac{1}{2},\tfrac{1}{2})$; $27.18(23)$. **(** **B** **)** Spike-frequency adaptation $(\tfrac{1}{10},1,\tfrac{1}{1000},5)$; $29.13(29)$. **(** **C** **)** Rebound spiking $(\tfrac{1}{50},2,\tfrac{1}{100},\tfrac{1}{5})$; $7.84(8)$. **(** **D** **)** Accommodation $(\tfrac{3}{25},3,\tfrac{1}{50},\tfrac{2}{5})$; $3.09 (3)$. **(** **E** **)** Spike latency $(\tfrac{1}{10},0,\tfrac{1}{200},\tfrac{2}{5})$; $16.12(16)$. **(** **F** **)** Inhibition-induced spiking $(\tfrac{1}{50},-1,\tfrac{1}{500},\tfrac{2}{5})$; $15.75(16)$

The rate-reduced neuron model (RNM) preserves the dynamical features of the full model (SNM) and reproduces all previous example spiking patterns (Fig. 5).

### Frequency Response of the Reduced Model

Analogously to Sect. 2.3, we now study the response of the rate-reduced model (RNM) to sinusoidal input

17 $$ u(t)= \varphi\cos \biggl(\frac{\omega\pi t}{1000}+\vartheta \biggr). $$

Under the assumption that

18 $$ \kappa u(t)-a(t)\leq\theta\quad\forall t, $$

the explicit solution of (RNM) is given by

19 $$ a(t)=-\varepsilon(1-\kappa) \int_{-\infty}^{t} e^{-\varepsilon (t-\tau)}u(\tau) \,\mathrm{d} \tau, $$

cf. (9). Inserting the input (17) into (19) yields

20 $$ \begin{aligned} \kappa u(t)-a(t)&= \kappa\varphi\cos \biggl(\frac{\omega\pi t}{1000}+\vartheta \biggr)+\varepsilon(1-\kappa)\varphi \int _{-\infty}^{t} e^{-\varepsilon(t-\tau)}\cos \biggl( \frac{\omega\pi \tau}{1000}+\vartheta \biggr) \,\mathrm{d}\tau \\ &=G(\omega)\frac{\varphi}{2}e^{(\frac{\omega\pi t}{1000}+\vartheta )i}+\overline{G(\omega)} \frac{\varphi}{2}e^{-(\frac{\omega\pi t}{1000}+\vartheta)i} \\ &=\bigl\lvert G(\omega)\bigr\rvert \varphi\cos \biggl(\frac{\omega\pi t}{1000}+ \vartheta+\arg \bigl(G(\omega) \bigr) \biggr), \end{aligned} $$

where the frequency response $G\colon\mathbb{R}_{\geq0}\mapsto \mathbb{C}$ is given by

21 $$ G(\omega)=\kappa+\frac{\varepsilon(1-\kappa)}{\varepsilon+\frac {\omega\pi i}{1000}}. $$

It follows that for the rate-reduced model (RNM) receiving harmonic input (17) we have

22 $$ S \bigl(\kappa u(t)-a(t)-\theta \bigr)=0 \quad\forall t \quad \text{if and only if} \quad\bigl\lvert G(\omega)\bigr\rvert \varphi\leq\theta. $$

**Fig. 6:**
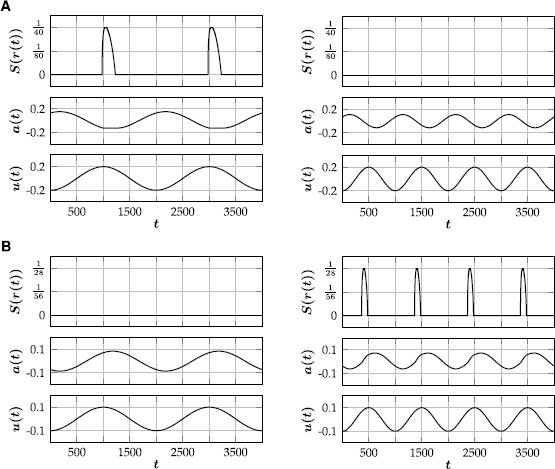
Responses of the rate-reduced model (RNM) to periodic input. Top traces show the firing rate with $r(t)= \kappa u(t)-a(t)-\theta$. **(** **A** **)** For $\kappa=\frac{1}{10}$ the model acts as a low-pass filter. Input with an amplitude of $\varphi=\frac{1}{5}$ yields a response in the firing rate for $\omega=1$, whereas the firing rate remains zero for $\omega=2$. In the former case, the integral of the spiking rate during one period is approximately 4.55, while there are 5 spikes per period in the full model (Fig. 4A). **(** **B** **)** For $\kappa=2$, the reduced model acts as a high-pass filter. Input with amplitude $\varphi=\tfrac{1}{10}$ elicits a firing rate response for $\omega=2$, whereas a lower input frequency of $\omega=1$ does not. In the former case, the integral of the spiking rate during one period is approximately 3.14, while there are 3 spikes per period in the full model (Fig. 4B). In both examples, $(\theta,\varepsilon,\gamma )= (\tfrac{1}{7},\tfrac{1}{200},2 )$

Because we neglected the transient corresponding to the convergence from fixed point to limit cycle in the rate-reduced model (RNM), the inequality in (22) defines a clear ‘spiking condition’. The modulus of the frequency response (21) is given by

23 $$ \bigl\lvert G(\omega)\bigr\rvert =\sqrt{\frac{\varepsilon^{2}+\kappa^{2} (\frac{\pi\omega}{1000} )^{2}}{\varepsilon^{2}+ (\frac{\pi \omega}{1000} )^{2}}}, $$

and $\lvert G\rvert$ therefore is strictly decreasing if and only if $\lvert\kappa\rvert\leq1$, and increasing otherwise. Revisiting the examples from Sect. 2.3 (Fig. 4), we have

24 $$ \bigl\lvert G(1)\bigr\rvert \varphi= 0.1696\ldots>\theta>0.1255\ldots=\bigl\lvert G(2)\bigr\rvert \varphi, $$

for $(\kappa,\varepsilon,\theta,\varphi )= (\frac {1}{10},\frac{1}{200},\frac{1}{7},\frac{1}{5} )$, and

25 $$ \bigl\lvert G(1)\bigr\rvert \varphi= 0.1359\ldots< \theta< 0.1684\ldots=\bigl\lvert G(2)\bigr\rvert \varphi, $$

for $(\kappa,\varepsilon,\theta,\varphi )= (2,\frac {1}{200},\frac{1}{7},\frac{1}{10} )$. Indeed, the rate-reduced model (RNM) reproduces the examples of the full model both qualitatively and quantitatively (Fig. 6). When the rate-reduced model (RNM) is incorporated into existing neural field models, the frequency response of the reduced model can be used to tune the individual temporal filter properties of the different neuronal populations.

### The Firing Rate Function

Since our neuron model (SNM) is a map, the period *P* of its limit cycle lies in $\mathbb{N}$ for all positive suprathreshold drives *ς*. Therefore, the spiking rate function (5) is staircase-like, with points of discontinuity whenever $P\to P+1$. Let $\lbrace\varsigma_{1},\varsigma_{2},\ldots\rbrace$ denote the set of all points of discontinuity of the firing rate function in decreasing order. For $\varsigma\geq\varsigma_{1}=1$ the ‘reset potential’ $v=-50$ in (FSS) is immediately mapped to a non-negative number, and the neuron is therefore spiking at its maximal frequency of once in three iterations. Similarly, the voltage stays in the left interval for two iterations and the neuron is spiking once in four iterations for $\varsigma_{1}>\varsigma\geq\varsigma_{2}=\frac {1}{2}(5-\sqrt{17})$. In general, at $\varsigma_{k}$, there is a jump discontinuity of size

26 $$ \lim_{\varsigma\rightarrow\varsigma_{k}^{+}}S(\varsigma)-\lim_{\varsigma\rightarrow\varsigma_{k}^{-}}S( \varsigma)=\frac {1}{(k+2)(k+3)},\quad\text{with } S(\varsigma_{k})= \frac{1}{k+2}. $$

The firing rate of the fast subsystem (FSS) can therefore be written as

27 $$ S(\varsigma)=\sum_{k=1}^{\infty} \frac{H(\varsigma -\varsigma_{k})}{(k+2)(k+3)}, $$

where *H* is the Heaviside step function and

28 $$ \lim_{k\to\infty}\varsigma_{k}=0. $$

In large neuronal networks, it is often assumed that the spiking thresholds of the individual neurons are randomly distributed. This ensures heterogeneity and models intrinsic interneuronal differences or random input from outside the network. If we add Gaussian noise to the threshold parameter *θ* in (SNM), it is natural to define an expected firing rate $\langle S\rangle\colon\mathbb{R}\mapsto\mathbb{R}$, given by

29 $$ \bigl\langle S(\varsigma)\bigr\rangle =\frac{1}{\sqrt{2\pi\sigma^{2}}} \int _{-\infty}^{\infty}e^{\frac{-w^{2}}{2\sigma^{2}}}S(\varsigma +w) \, \mathrm{d}w, $$

where $\sigma^{2}$ is the variance of the noise. Using (27), we can rewrite (29) as

30 $$ \bigl\langle S(\varsigma)\bigr\rangle =\frac{1}{6}+\sum _{k=1}^{\infty }\frac{\operatorname{erf}(\frac{\varsigma-\varsigma_{k}}{\sqrt{2\sigma ^{2}}} )}{2(k+2)(k+3)}, $$

where erf denotes the error function. While $S(\varsigma)$ can readily be computed for any $\varsigma\in\mathbb{R}$ and we derived a concise expression for the expected firing rate, the infinite sum (30) cannot easily be evaluated. For this reason, we approximate $\langle S(\varsigma)\rangle$ by a finite sum of the form

31 $$ \frac{1}{6}+\frac{1}{6N}\sum _{i=1}^{N}\operatorname{erf}\biggl(\frac {\varsigma-\nu_{i}}{\chi_{i}} \biggr), $$

for some fixed $N\in\mathbb{N}$ and constants $\nu_{i},\chi_{i}\in \mathbb{R}$, which are chosen by (numerically) minimizing

32 $$ \Biggl\lVert \frac{1}{\sqrt{2\pi\sigma^{2}}} \int_{-\infty }^{\infty}e^{\frac{-w^{2}}{2\sigma^{2}}}S(\varsigma+w) \, \mathrm{d}w-\frac{1}{6}-\frac{1}{6N}\sum _{i=1}^{N}\operatorname{erf}\biggl(\frac{\varsigma-\nu_{i}}{\chi_{i}} \biggr) \Biggr\rVert _{2}. $$

**Fig. 7:**
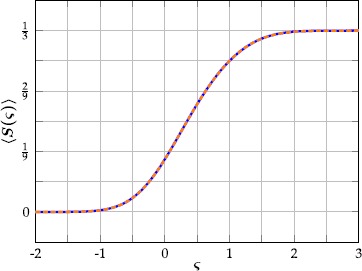
Expected firing rate for a noise level of ${\sigma^{2}=\tfrac{1}{4}}$. Shown are a numerical integration of (29) (*blue*) and its approximation (31) for $N=2$ and $(\nu_{1},\nu_{2},\chi_{1},\chi_{2} )= (0.0335, [4] 0.7099,0.6890,0.8213 )$ (*orange*)

For large noise levels $\sigma^{2}$, the average firing rate (29) has a sigmoidal shape and can be very well approximated with a small value of *N* (Fig. 7).

## Augmenting Neural Fields

When large populations of neurons are modeled by networks of individual, interconnected cells, the high dimensionality of state and parameter spaces makes mathematical analysis intractable and numerical simulations costly. Moreover, large network simulations provide little insight into global dynamical properties. A popular modeling approach to circumventing the aforementioned problems is the use of neural field equations. These models aim to describe the dynamics of large neuronal populations, where spikes of individual neurons are replaced by (averaged) spiking rates and space is continuous. Another advantage of neural fields is that they are often well suited to model experimental data. In brain slice preparations, spiking rates can be measured with an extracellular electrode, while intracellular recordings are much more involved. Furthermore, the most common clinical measurement techniques of the brain, electroencephalography (EEG) and functional magnetic resonance imaging (fMRI), both represent the average activity of large groups of neurons and may therefore be better modeled by population equations. The first neural field model can be attributed to Beurle [1], however, the theory really took off with the work of Wilson and Cowan [2, 3], Amari [5, 6], and Nunez [4].

In ‘classical’ neural field models the firing rate of a neuronal population is assumed to be given by its instantaneous input, which is only valid for tonically spiking neurons. With the help of our rate-reduced model (RNM), it is straightforward to augment existing neural field models with more complex spiking behavior. As an example, we will look at the following two-population model on the one-dimensional spatial domain $\varOmega=(-1,1)$:

ANF $$ \begin{aligned} \biggl(1+\frac{1}{\alpha_{1}} \frac{\partial}{\partial t} \biggr) u_{1}(t,x) &= \int_{-1}^{1}J_{11} \bigl(x,x' \bigr)S_{1} \bigl(r_{1}\bigl(t,x'\bigr) \bigr)\\ &\quad {}+J_{12} \bigl(x,x' \bigr)S_{2} \bigl(r_{2}\bigl(t,x'\bigr) \bigr) \,\mathrm{d}x', \\ \biggl(1+\frac{1}{\varepsilon_{1}}\frac{\partial}{\partial t} \biggr) a_{1}(t,x) &= -(1-\kappa_{1})u_{1}(t,x)+\gamma_{1} S_{1} \bigl(r_{1}(t,x) \bigr), \\ \biggl(1+\frac{1}{\alpha_{2}}\frac{\partial}{\partial t} \biggr) u_{2}(t,x) &= \int_{-1}^{1}J_{21} \bigl(x,x' \bigr)S_{1} \bigl(r_{1}\bigl(t,x'\bigr) \bigr)\\ &\quad {}+J_{22} \bigl(x,x' \bigr)S_{2} \bigl(r_{2}\bigl(t,x'\bigr) \bigr) \,\mathrm{d}x', \\ \biggl(1+\frac{1}{\varepsilon_{2}}\frac{\partial}{\partial t} \biggr) a_{2}(t,x) &= -(1-\kappa_{2})u_{2}(t,x)+\gamma_{2} S_{2} \bigl(r_{2}(t,x) \bigr), \end{aligned} $$

where, as before,

33 $$ r_{i}(x,t)=\kappa_{i} u_{i}(t,x)-a_{i}(t,x)- \theta_{i}, $$

for $i\in\lbrace1,2\rbrace$. The differential operators in the left-hand side of the integral equations in (ANF) model exponentially decaying synaptic currents with decay rate $\alpha_{i}$. The connectivity $J_{ij} (x,x' )$ measures the connection strength from neurons of population *j* and position $x'$ to neurons of population *i* and position *x*. The connectivity kernels $J_{ij}\colon \overline{\varOmega}\times\overline{\varOmega}\mapsto\mathbb{R}$ are assumed to be isotropic and given by

34 $$ J_{ij}\bigl(x,x'\bigr)=\rho_{j} \eta_{ij}e^{-\mu_{ij}\lvert x-x'\rvert}, $$

where $\rho_{j}$ is the density of neurons of type *j*, $\eta_{ij}$ is the maximal connection strength, and $\mu_{ij}$ is the spatial decay rate of the connectivity. Both firing rate functions $S_{i}\colon\mathbb{R}\mapsto\mathbb{R}$ are chosen to approximate the expected firing rate of Rulkov neurons (SNM) with a noise level of $\sigma^{2}=\frac{1}{4}$ (Fig. 7),

35 $$ S_{1}(\varsigma)=S_{2}(\varsigma)=\frac{1}{6}+ \frac{1}{12}\operatorname{erf}\biggl(\frac{\varsigma-0.0335}{0.6890} \biggr)+\frac{1}{12}\operatorname{erf}\biggl(\frac{\varsigma-0.7099}{0.8213} \biggr). $$

**Fig. 8:**
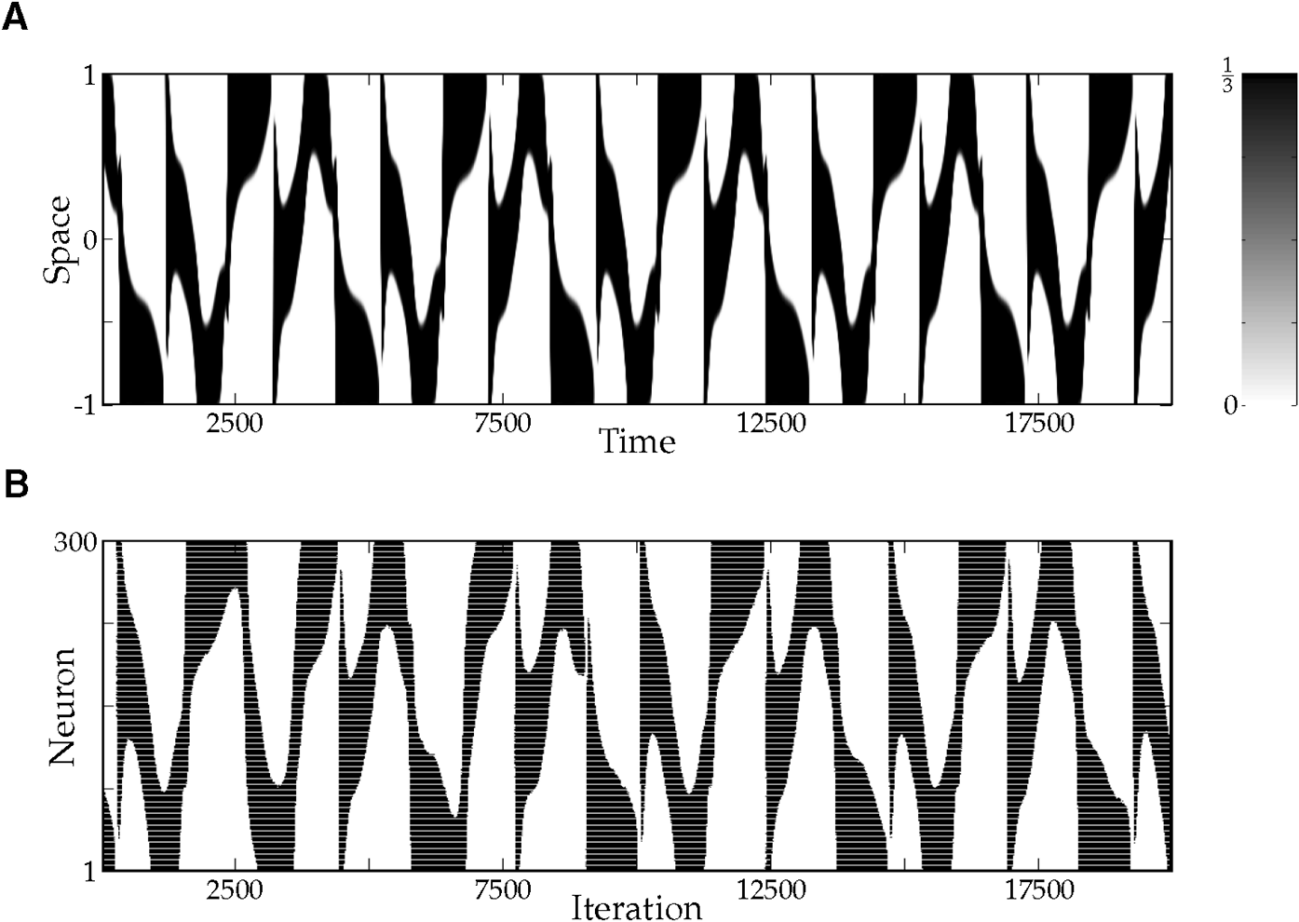
Spatio-temporal spiking patterns. **(** **A** **)** Simulation of the augmented neural field (ANF) with parameter values given in Table 1. Shown is the firing rate $S_{1} (\kappa_{1} u_{1}(t,x)-a_{1}(t,x)-\theta_{1} )$ of the first population. **(** **B** **)** Simulation of a corresponding network of 300 excitatory and 300 inhibitory Rulkov neurons, all-to-all coupled via simple exponential synapses. Both populations are equidistantly placed on the interval $[-1,1]$. Uncorrelated (in space and time) Gaussian noise with variance $\sigma^{2}=\tfrac{1}{4}$ is added to the threshold parameter of each neuron. Shown is the spiking activity of the excitatory population. Each spike is denoted by a black dot

We conclude this section with a simulation of (ANF) for a particular parameter set (Table 1), which illustrates that our augmented neural field can generate interesting spatiotemporal behavior that closely resembles spiking patterns of a network of Rulkov neurons (SNM) with corresponding parameter values (Fig. 8). In the Rulkov network, synaptic input to neuron *i* is given by

36 $$ u^{(i)}_{n+1} = (1-\alpha_{i})u^{(i)}_{n}+ \alpha_{i}\sum_{j=1}^{N} c_{ij}s^{(j)}_{n}, $$

where *N* denotes the total number of neurons in the network, and $c_{ij}$ is the connection strength from neuron *j* to neuron *i*. To match the parameters in Table 1, we split the total population in two subpopulations of 300 neurons each, which are both equidistantly placed on the interval $[-1,1]$. Neurons within the same subpopulation share the same intrinsic parameters, and uncorrelated (in space and time) Gaussian noise is added to the threshold parameters. Finally, the connection strengths in the Rulkov network are given by

37 $$ c_{ij}=\eta_{{p_{i}}{p_{j}}}e^{-\mu_{{p_{i}}{p_{j}}}\lvert x_{i}-x_{j}\rvert}, $$

where $p_{i}$ and $x_{i}$ are the subpopulation and position of neuron *i*, respectively.

**Table 1:** **Parameter overview for the neural field** (**ANF**)

Parameter	$\alpha_{i}$	$\theta_{i}$	$\kappa_{i}$	$\varepsilon_{i}$	$\gamma_{i}$	$\rho_{i}$	$\eta_{i1}$	$\eta_{i2}$	$\mu_{i1}$	$\mu_{i2}$
Population 1	$\frac{1}{20}$	$\frac{1}{2}$	2	$\frac{1}{1000}$	5	150	$\frac{2}{3}$	$-\frac{1}{3}$	4	1
Population 2	$\frac{1}{10}$	$\frac{4}{5}$	$\frac{1}{10}$	$\frac{1}{100}$	2	150	$\frac{11}{15}$	$-\frac{11}{30}$	$\frac{17}{4}$	$\frac{11}{10}$

## Discussion

This paper presents a simple rate-reduced neuron model that is based on a variation of the Rulkov map (Rulkov [14], Rulkov et al. [15]), and can be used to incorporate a variety of non-trivial spiking behavior into existing neural field models.

The modified Rulkov map (SNM) is a phenomenological, two-dimensional single neuron model. The isolated dynamics of its fast time scale either generates a stable limit cycle, mimicking spiking activity, or a stable fixed point, corresponding to a neuron at rest (Fig. 1). The slow time scale of the Rulkov map acts as a dynamic spiking threshold and emulates the combined effect of slow recovery processes. The modified Rulkov map can mimic a wide variety of spiking patterns, such as spike-frequency adaptation, postinhibitory rebound, phasic spiking, spike accommodation, spike latency and inhibition-induced spiking (Fig. 2). Furthermore, the model can be used to model neuronal filter properties. Depending on how external input is applied to the model, it can act as either a high-pass or low-pass filter (Figs. 3 and 4).

The rate-reduced model (RNM) is derived heuristically and given by a simple one-dimensional differential equation. On the single cell level, the rate-reduced model closely mimics the spiking dynamics (Fig. 5) and filter properties (Fig. 6) of the full spiking neuron model. While a close approximation of the (expected) firing rate of Rulkov neurons (Fig. 7) is needed to mimic their behavior quantitatively, the types of qualitative dynamics of the rate-reduced model do not depend on the exact choice of firing rate function.

Due to its simplicity, it is straightforward to add the rate-reduced model to existing neural field models. In the resulting augmented equations, parameters can be chosen according to the spiking behavior of a single isolated cell. In our particular example (ANF), the emerging spatiotemporal pattern closely resembles the dynamics of the corresponding spiking neural network (Fig. 8). We believe that this is an elegant way to add more biological realism to existing neural field models, while simultaneously enriching their dynamical structure.

### Conclusions

We used a variation of a simple toy model of a spiking neuron (Rulkov [14], Rulkov et al. [15]) to derive a corresponding rate-reduced model. While being purely phenomenological, the model could mimic a wide variety of biologically observed spiking behaviors, yielding a simple way to incorporate complex spiking behavior into existing neural field models. Since all parameters in the resulting augmented neural field equations have a representative in the spiking neuron network (and vice versa), this greatly simplifies the otherwise often problematic translation from results obtained by neural field models back to biophysical properties of spiking networks. An example demonstrated that the augmented neural field equations can produce spatiotemporal patterns that cannot be generated with corresponding ‘classical’ neural fields.
